# Supplementary material for: A Rare Variant in MDH2 (rs111879470) Is Associated with Predisposition to Recurrent Breast Cancer in an Extended High-Risk Pedigree
Source: Cancers (Basel). 2023 Dec 15;15(24):5851. doi: 10.3390/cancers15245851 (PMC10741671; doi:10.3390/cancers15245851)
Supplement: Supplementary file 1 [file cancers-15-05851-s001.zip › cancers-2619236-supplementary.pdf]

Supplementary Material

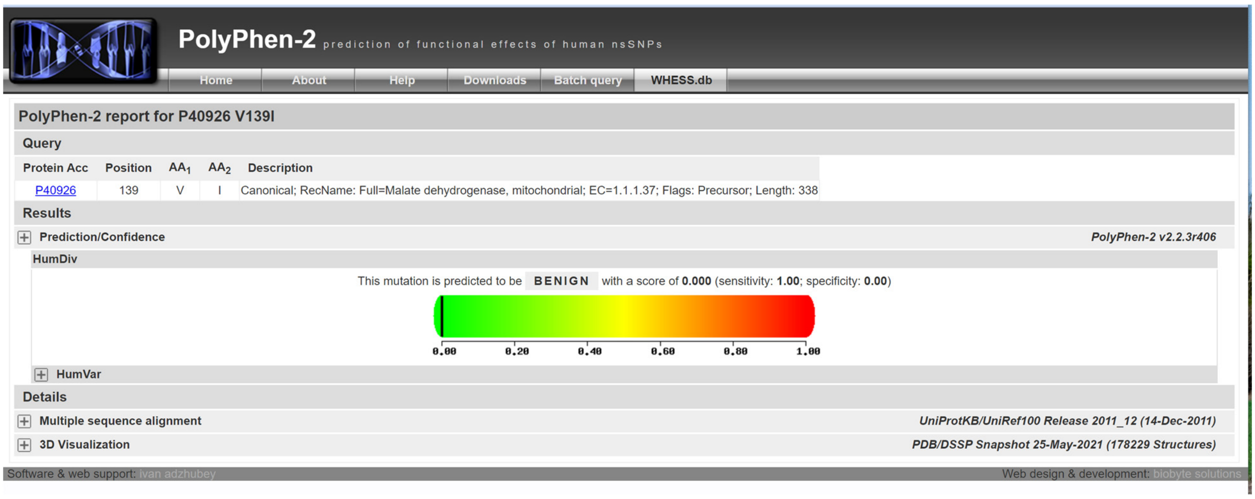

Figure S1. PolyPhen2 prediction of pathogenicity of the MDH2 V139I variant.

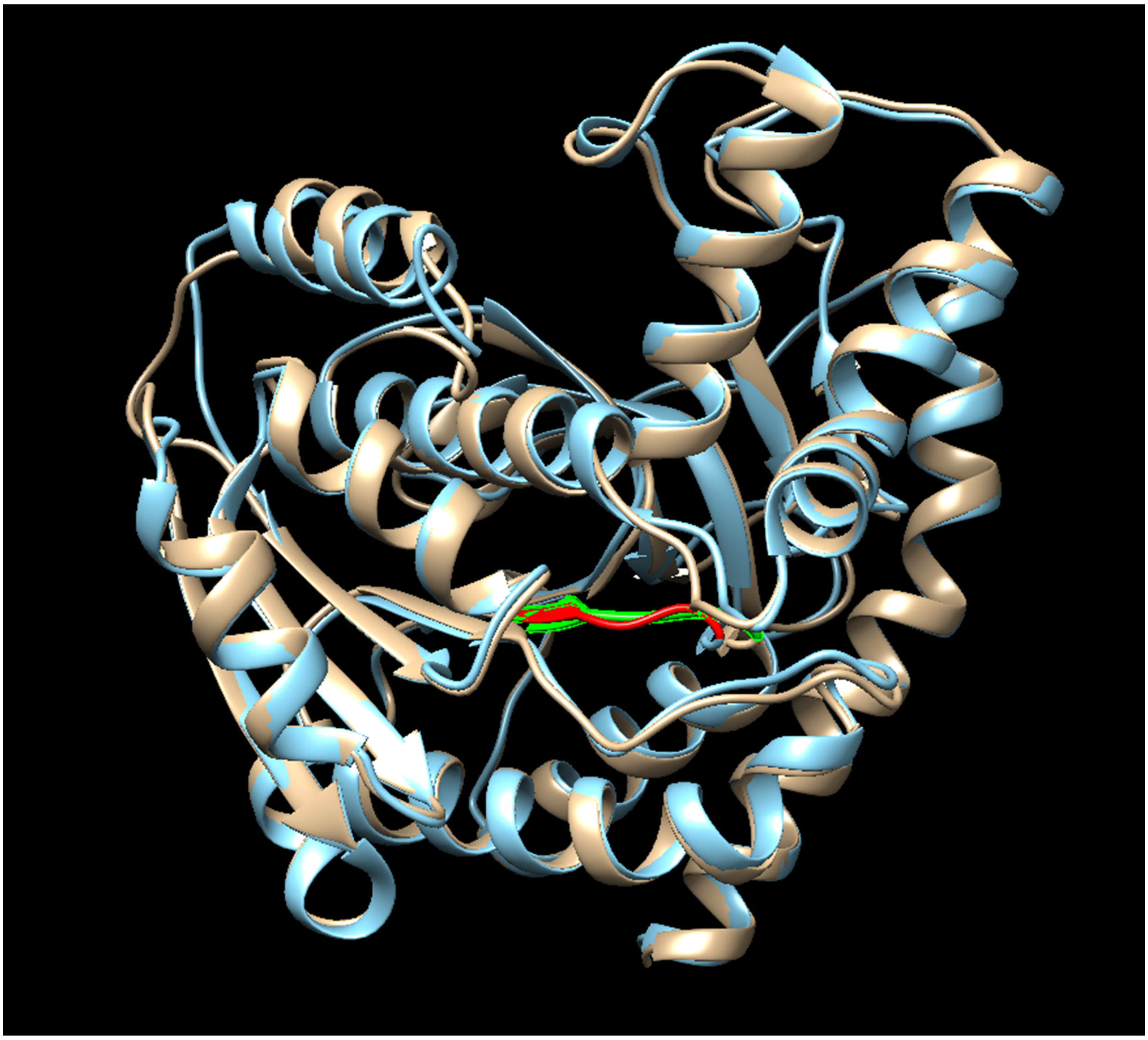

Figure S2. Comparison of the 3D (predicted) structures of the WT (in gold) and MUT (in blue) MDH2 proteins, which show an agreement RMSD between 318 pruned atom pairs is 0.644 angstroms; (across all 338 pairs: 1.202). The regions of the mutation is highligted in red (MUT) and green (WT).

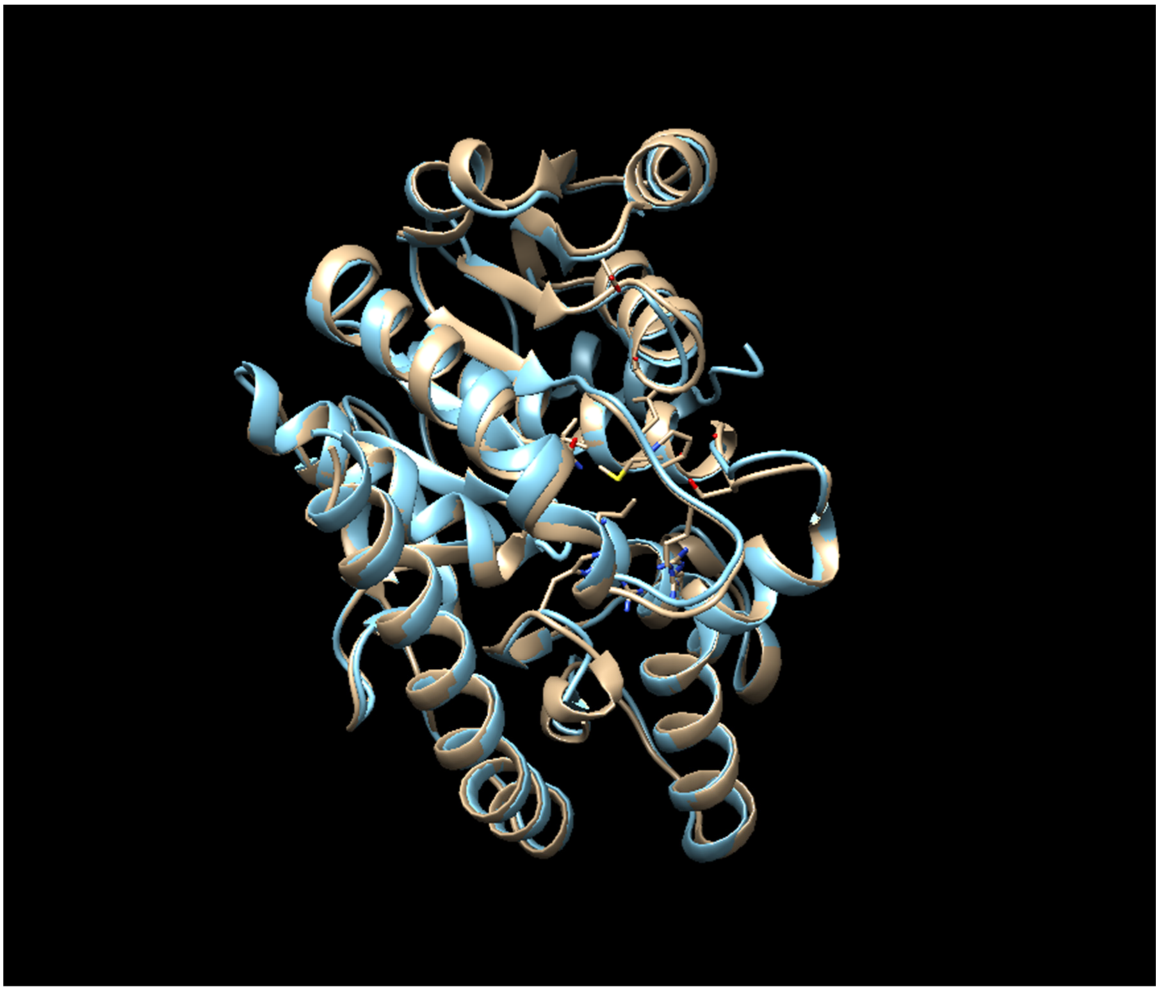

Figure S3. Comparison of the I-TASSER predicted structure for the WT of MDH2 with the experimental and AlphaFold Predicted one.

Comparison of the I-TASSER predicted structure (gold) for the WT of MDH2 with the experimental (blue) RMS for 311 pruned pairs 0.565 A, for all 314 pairs 0.652 A.

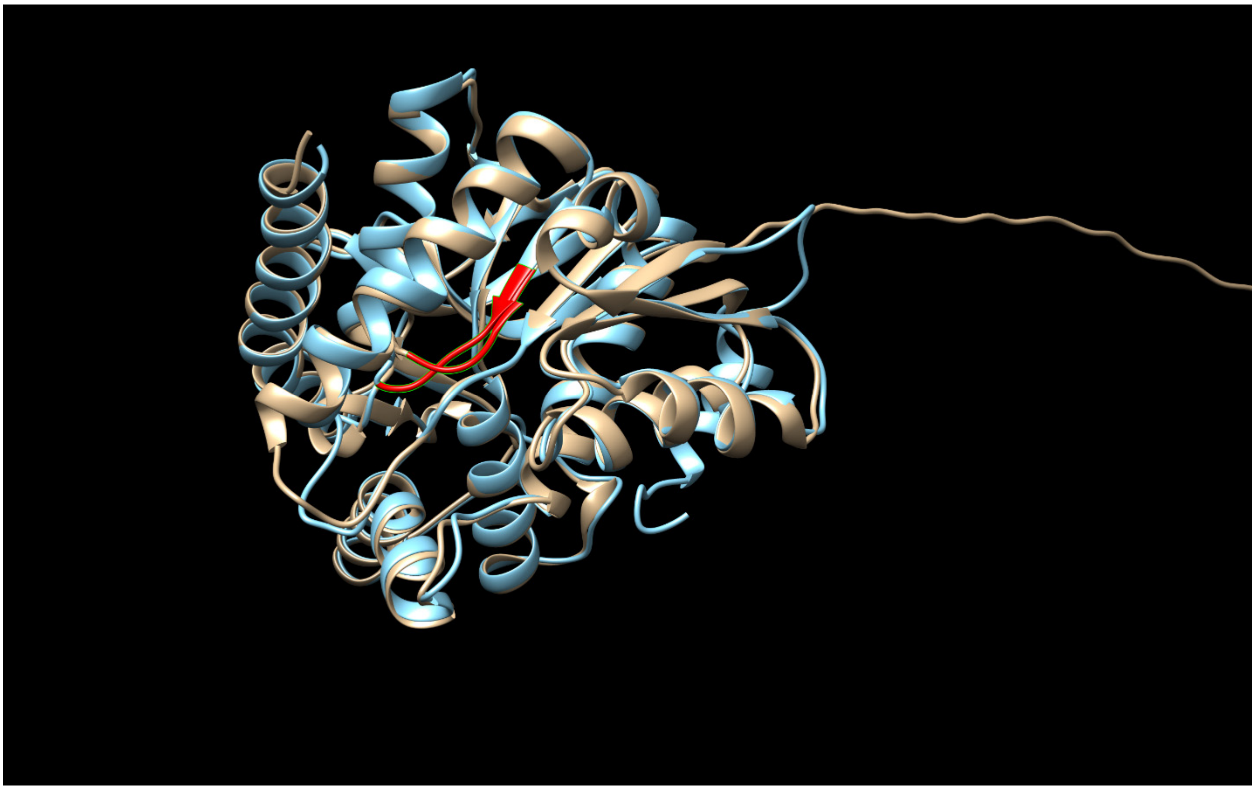

MDH2 AlphaFold (brown) vs I-TASSER (blue), in RED the region of the V139I mutation RMSD between 297 pruned atom pairs is 0.541 angstroms; (across all 338 pairs: 10.851).

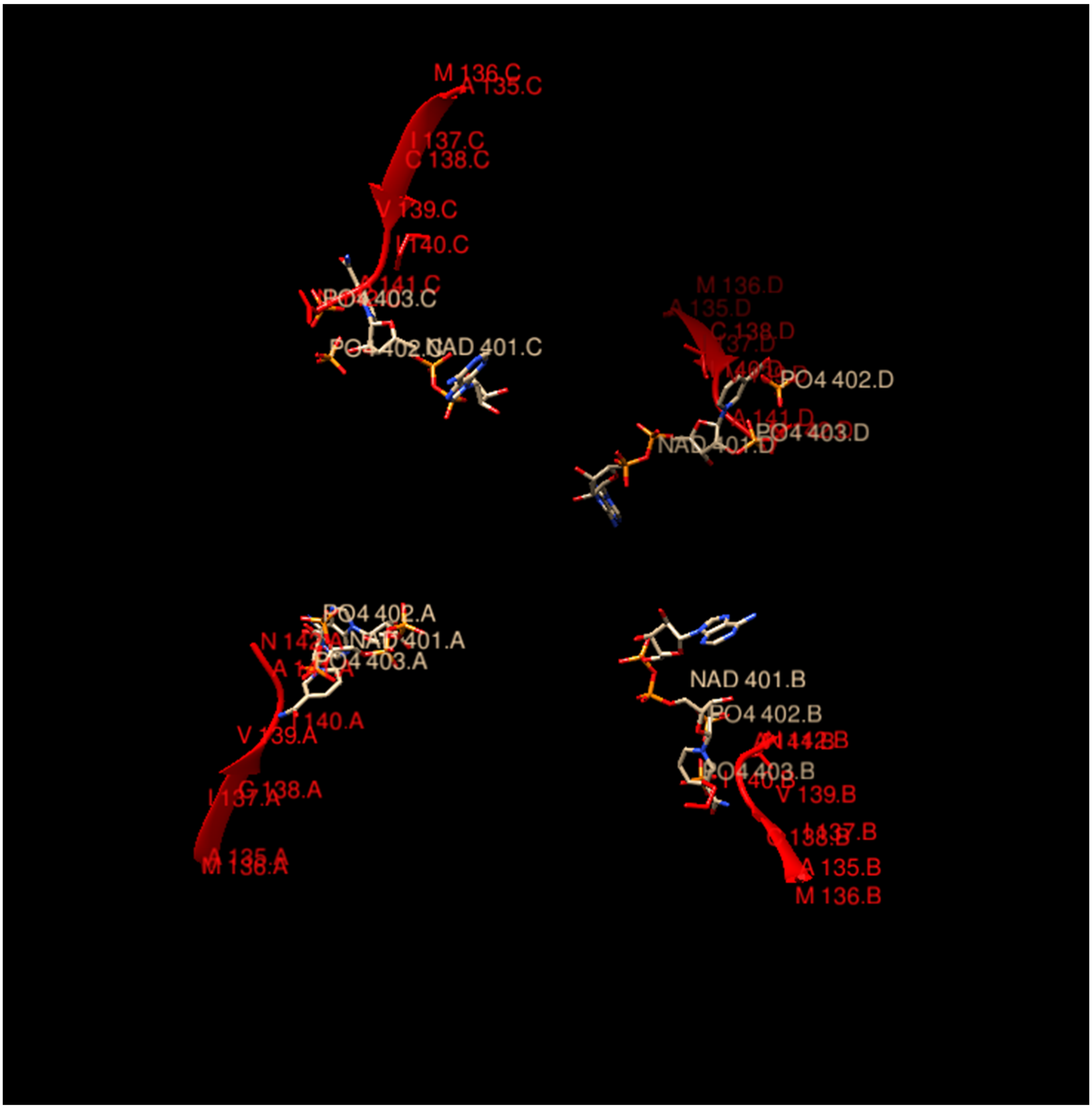

Figure S4. Binding of NAD to MDH2: Note that NAD binds exactly in the region of the mutation V139I.. https://www.ebi.ac.uk/pdbe/entry/search/index/?searchParams=%7B%22q_uniprot%22:%5B%7B%22value%22:%22P08249%22,%22condition1%22:%22AND%22,%22condition2%22:%22Contains%22%7D,%7B%22value%22:%22P40926%22,%22condition1%22:%22OR%22,%22condition2%22:%22Contains%22%7D%5D,%22resultState%22:%7B%22tabIndex%22:0,%22paginationIndex%22:1,%22perPage%22:%2210%22,%22sortBy%22:%22Sort%20by%22%7D%7D.
